# Modeling Soil Temperature for Different Days Using Novel Quadruplet Loss-Guided LSTM

**DOI:** 10.1155/2022/9016823

**Published:** 2022-02-17

**Authors:** Xuezhi Wang, Wenhui Li, Qingliang Li, Xiaoning Li

**Affiliations:** ^1^College of Computer Science and Technology, Jilin University, Changchun 130012, China; ^2^Symbol Computation and Knowledge Engineer of Ministry of Education, Jilin University, ChangChun, Jilin Province, China; ^3^School of Computer Science and Technology, Changchun Normal University, Changchun 130032, China

## Abstract

Soil temperature (*T*_*s*_), a key variable in geosciences study, has generated growing interest among researchers. There are many factors affecting the spatiotemporal variation of *T*_*s*_, which poses immense challenges for the *T*_*s*_ estimation. To enrich processing information on loss function and achieve better performance in estimation, the paper designed a new long short-term memory model using quadruplet loss function as an intelligence tool for data processing (QL-LSTM). The model in this paper combined the traditional squared-error loss function with distance metric learning between the sample features. It can zoom analyze the samples accurately to optimize the estimation accuracy. We applied the meteorological data from Laegern and Fluehli stations at 5, 10, and 15 cm depth on the 1st, 5th, and 15th day separately to verify the performance of the proposed soil temperature estimation model. Meanwhile, this paper inputs the variables into the proposed model including radiation, air temperature, vapor pressure deficit, wind speed, air pressure, and past *T*_*s*_ data. The performance of the model was tested by several error evaluation indices, including root mean square error (RMSE), mean absolute error (MAE), Nash-Sutcliffe model efficiency coefficient (NS), Willmott Index of Agreement (WI), and Legates and McCabe index (LMI). As the test results at different soil depths show, our model generally outperformed the four existing advanced estimation models, namely, backpropagation neural networks, extreme learning machines, support vector regression, and LSTM. Furthermore, as experiments show, the proposed model achieved the best performance at the 15 cm depth of soil on the 1st day at Laegern station, which achieved higher WI (0.998), NS (0.995), and LMI (0.938) values, and got lower RMSE (0.312) and MAE (0.239) values. Consequently, the QL-LSTM model is recommended to estimate daily *T*_*s*_ profiles estimation on the 1st, 5th, and 15th days.

## 1. Introduction

Soil temperature (*T*_*s*_) is a main physical variable of the land surface, which has a direct influence on the atmosphere [1]. Relevant fields including geoscience and forestry application aspects have drawn attention from researchers [2, 3]. In principle, all the interactions in terrestrial ecosystems are companied by *T*_*s*_ variations since they involve energy exchanges. *T*_*s*_ is an essential factor for growing crops that can facilitate the development of the root system by impacting microbial activity, soil decomposition, and fluidity of soil water [4]. In addition, the death of animals and plants produces plenty of carbon substrates and a high volume of greenhouse gases in the soil. Consequently, it results in an increase in *T*_*s*_, thus expediting carbon dioxide emission to the atmosphere [5]. Therefore, accurate *T*_*s*_ monitoring is crucial for agricultural management and atmosphere environment forecast. However, *T*_*s*_ data in most areas is still measured by using traditional sensors, and the *T*_*s*_ data cannot be collected at different depths [6].

Therefore, it can be used to solve some problems in different fields for the study of *T*_*s*_ estimation. The essential environmental factors have a great influence on the accuracy of *T*_*s*_ estimation. At present, *T*_*s*_ is mainly predicted by methods based on physical models and data-driven methods. The physical method is based on the heat conduction model to estimate *T*_*s*_ [7]. Meanwhile, the method is greatly affected by related physical parameters and the scale problem [8].

The data-driven methods can explore the internal relationship between *T*_*s*_ and the surrounding environmental factors for *T*_*s*_ estimation. At present, several predictive models based on machine learning methods are used for estimating *T*_*s*_ [9–14]. For example, ANN is composed of a complex network structure that imitates the structure and function of the brain's neural network, and it has powerful data processing capabilities. Bilgili applied the multilayer perceptron (MLP) model to adequately describe *T*_*s*_ distribution at a monthly temporal scale from meteorological data [15]. Kisi et al. used three machine learning models to estimate monthly *T*_*s*_ at the soil depth of 5 cm and 10 cm, respectively, and verified the predictive performance of radial basis neural networks performed better than that of generalized regression neural networks and multilayer perceptron models [9]. But generalized regression neural networks had the better performance for deeper depth (50 cm and 100 cm). Kisi et al. applied ANN-based models to predict long-term *T*_*s*_ at a monthly temporal scale, and they found that genetic programming generated the best performance with the meteorological data [16]. Zeynoddin et al. applied a multilayer perceptron (MLP) model to describe daily *T*_*s*_ distribution at three soil depths (5, 10, and 20 cm) from past measurements of *T*_*s*_ [17]. Samadianfard et al. processed the meteorological data such as Ta, W, RH, Rs, Sunshine hours (Sh), and air pressure (Ap) and integrated ANN-based models separately to predict *T*_*s*_ at a monthly temporal scale [18]. Mehdizadeh et al. noted that machine learning models combined with time series models performed better performance than the predictive models based on the single machine learning method or single time series method for predicting *T*_*s*_ at a daily temporal scale [19]. Moazenzadeh et al. proposed SVR with krill herd algorithm (SVR-KHA) method in modeling *T*_*s*_ estimation at different depths (5, 10, 20, 30, 50, and 100 cm), which achieved the best performance, compared to SVR and SVR with firefly algorithm (SVR-FA) [11]. Delbari et al. proposed an SVR-based model to compute daily *T*_*s*_ at three depths (5, 30, and 100 cm) in Iran [12]. The ELM network featured by a single hidden layer could improve the learning speed and accuracy of the algorithm and can model the accurate *T*_*s*_. Nahvi et al. used the improved ELM model on daily *T*_*s*_ estimation based on the self-adaptive evolutionary method and verified the improved predictive model can estimate the adequate *T*_*s*_ [20]. Sanikhani et al. tested the data from the Mersin station, and the results that show the performance of ELM has the best predictive performance than other predictive models. [14]. Feng et al. tested the loess plateau data with ELM and random forests (RF) and ANN-based models showed that ELM had the better performance for estimation *T*_*s*_ of half-hourly at different soil depths [13].

As a time loop neural network containing complex neural network modules, LSTM is used in this paper to solve long-term dependence problems, which can effectively alleviate gradient vanishing through the extraction of required features by the gate control unit. LSTM network [21] can learn long-term and short-term behaviors, and it has seen application in vast areas. By integrating LSTM and SVR, Guo et al. significantly improved the prediction accuracy of abnormal passenger flow fluctuations [22]. In hydrology, Zhang et al. designed a novel LSTM model with the dropout scheme to estimate the depth of the water table [23]. In the atmosphere field, Qing et al. estimated the solar irradiance based on the LSTM network [24]; the results showed the method could avoid the overfitting of the model. Li et al. designed a new GANs-LSTM model and noted that it could serve as an alternative method to estimate *T*_*s*_ [25].

This article focuses on the following issues. First, we select the environmental factors which will affect *T*_*s*_ estimation. *T*_*s*_ memory can help the predictive model “remember” a warm or cold condition when the anomaly is forgotten by the atmosphere forcing. In addition, recent literature reviews have revealed that the input for prediction models is either the past measurements of *T*_*s*_ or other meteorological information (Ta, W, RH, Rs, Sh, and Ap). Assume that the prediction models are constructed using input combinations of past *T*_*s*_ and other meteorological information; how does the prediction model performance? The second question is about the construction of loss function in LSTM. The predictive model for *T*_*s*_ estimation is a regression predictive modeling problem that involves predicting a real-valued quantity. The loss function is crucial for optimizing the predictive model which could express the degree of difference between predicted and observed *T*_*s*_; meanwhile, it can optimize the predictive model by updating the weights. Recently, most previous studies in loss function of regression predictive model mainly focused on the distance metric learning between predicted values and real values [26–29]. However, the distance metric learning between the sample features (environmental factors) is usually ignored which has already been successfully applied to image processing [30–32]. To enrich information processing in loss function and further improve the estimation performance, how can we construct a novel loss function by combinations of distance metric learning? The last question is about timescale evaluating for *T*_*s*_ estimation. In previous studies, any evaluation at short-term *T*_*s*_ estimation (half-hourly, hourly, daily timescales) does not consider the timeliness of long-term *T*_*s*_ estimation. However, any evaluation at long-term *T*_*s*_ estimation (monthly timescale) does not include the information of *T*_*s*_ in a small timescale. An ideal decision-support tool for *T*_*s*_ estimation should provide a multifarious decision-making basis. How can we design a prediction scheme at the same timescale evaluation that provides not only the short-term decision-making basis but also the long-term decision-making basis?

**Figure 1:**
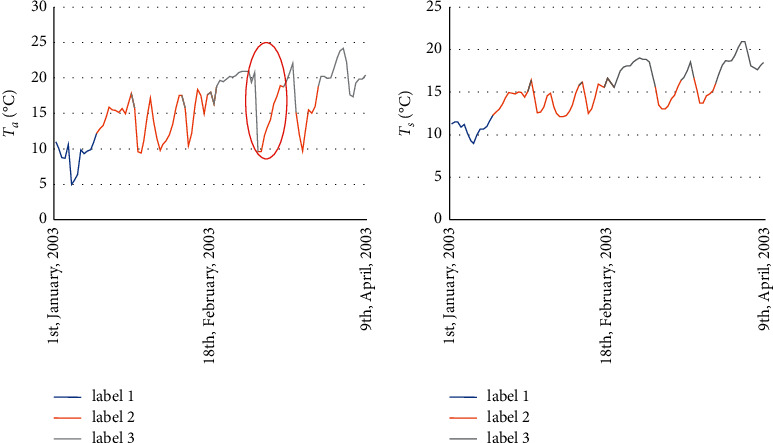
Variations of the daily air temperature (a) and soil temperature (b) at Laegern station (located in Switzerland) during 1st, January 2003–9th April 2003 (100 days).

This paper proposed a novel quadruplet loss function based on the LSTM network that combines traditional squared-error loss function with distance metric learning between the sample features, called QL-LSTM. The traditional squared-error loss function is usually applied to the predictive task with great accuracy. The current limitation of this loss function, however, involves the special variation on Ts based on different predictors. As shown in Figure 1, we have made labels according to *T*_*s*_ values. The *T*_*s*_ data which are in the same range are made the same label (the *T*_*s*_ data which are in the range of 8–12°C are labeled as “1”, the *T*_*s*_ data which are in the range of 12–16°C are labeled as “2”, and the *T*_*s*_ data which are in the range of 16–22°C are labeled as “3”). Meanwhile, Ta data are labeled as the same as *T*_*s*_ data. In Figure 1(a), we noticed that the *T*_*s*_ data with the same label are almost within a stable range. However, in the red ellipse, Figure 1(b), we observed that similar Ta values may have different labels (*T*_*s*_ data with similar Ta values may vary considerably). The data with this feature will prevent predictive models from accurately exploring the internal relationship between *T*_*s*_ and the surrounding environmental factors discovering. To address this problem, the idea of triplet loss [33] is considered in this paper. Triplet loss optimization allows the anchor and positive points to accumulate and therefore prevent the negative points and realize the similarity calculation of samples. This approach can enrich processing information of loss function and overcome the disadvantage of the traditional squared-error loss function and further improve the estimation performance.

The main three contributions of this research paper are summarized as follows:

1. As we know, the proposed method that combined traditional squared-error loss function with distance metric learning between the sample features is a new approach to be used for *T*_*s*_ estimation.
2. Daily-scale prediction scheme was designed to provide the multifarious decision-making basis and was used to estimate the *T*_*s*_ on the next 1st, 5th, and 15th day. To achieve this end, we input the meteorological and past *T*_*s*_ data to the estimation model.
3. Results showed that our QL-LSTM method outperformed the existing advanced methods in most cases.

## 2. Data and Methods

### 2.1. The Framework of Soil Temperature Estimation

**Figure 2:**
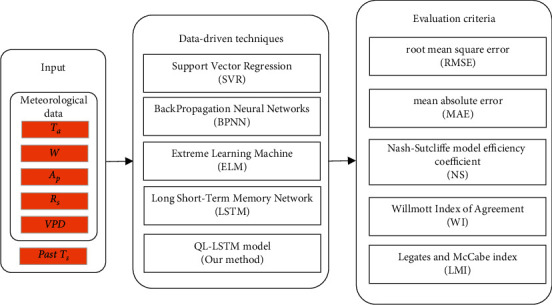
The flow chart of soil temperature estimation.

The corresponding meteorological data *T*_*s*_ as the input of our QL-LSTM model are obtained from FLUXNET at first. In the meantime, several other advanced models based on data-driven technology (SVR, BPNN, ELM, and LSTM) were considered in *T*_*s*_ estimation. Traditional squared-error loss function and distance metric learning between the sample features were integrated into our model for accurate exploration of the internal relationship between *T*_*s*_ and the surrounding environmental factors. Finally, the comparison of model performance is reflected by five evaluating indicators (RMSE, MAE, NS, WI, and LMI).  Figure 2 denoted the flow chart of soil temperature estimation.

### 2.2. Long Short-Term Memory (LSTM) Network

**Figure 3:**
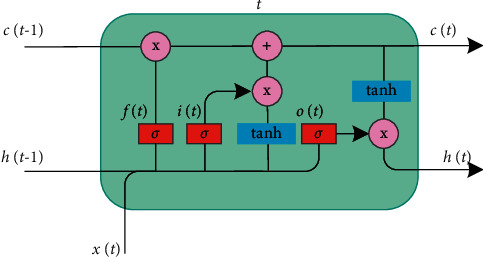
The internal structure of an LSTM cell.

LSTM can process and learn long-term dependence problems. Due to the characteristics of the LSTM network, we use it to explore the internal relationship between *T*_*s*_ and the surrounding predictors. LSTM controls the transmission state through the gating state, remembers what needs to be remembered, and forgets unimportant information.  Figure 3 shows the internal structure of an LSTM cell, and the calculation formula of the LSTM is as follows:

(1) $$\begin{array}{c} i\left(t\right)=\sigma \left(W_{ih}h\left(t-1\right)+W_{ix}x\left(t\right)+W_{ix}c\left(t-1\right)+b_{i}\right),\\ f\left(t\right)=\sigma \left(W_{fh}h\left(t-1\right)+W_{fx}x\left(t\right)+W_{fx}c\left(t-1\right)+b_{f}\right),\\ c\left(t\right)=f\left(t\right)⊗c\left(t-1\right)+i\left(t\right)⊗\;\tanh\left(W_{ch}h\left(t-1\right)+W_{cx}x\left(t\right)+b_{c}\right),\\ o\left(t\right)=\sigma \left(W_{oh}h\left(t-1\right)+W_{ox}x\left(t\right)+W_{oc}c\left(t\right)+b_{o}\right),\\ h\left(t\right)=o\left(t\right)⊗\;\tanh\left(c\left(t\right)\right),\\ \hat{y}\left(t\right)=W_{yh}h\left(t\right)+b_{o}, \end{array}$$

where *x*(*t*) is the input data, and $\hat{y}\left(t\right)$ is the output data; *i*(*t*), *f*(*t*), and *o*(*t*) denote the input gate, forget gate, and output gate; *c*(*t*) represents the unit status at the current moment; *h*(*t*) is the current output value; *σ*(·) and tanh(·) are the activation functions; *W* and *b* denote the weight matrix and bias term.

### 2.3. Triplet Loss

**Figure 4:**
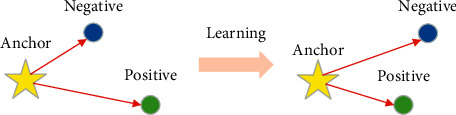
A visual representation of triplet loss.

Triplet loss is a significant “learning criterion” for optimizing the predictive models, which is applied for adjusting the weight parameters of predictive models, including anchor (Anchor) example, positive (Positive) example, and negative (Negative) example. The similarity calculation of the samples is realized through triplet loss learning, which makes the anchor-to-positive distance smaller than the anchor-to-negative distance. And Figure 4 denoted the visual representation of triplet loss.

Equation (2) expresses the objective function of triplet loss as follows:

(2) $$\begin{array}{c} \sum_{i}^{N}{\left[{\left\|f\left(x_{i}^{a}\right)-f\left(x_{i}^{p}\right)\right\|}_{2}^{2}-{\left\|f\left(x_{i}^{a}\right)-f\left(x_{i}^{n}\right)\right\|}_{2}^{2}+\alpha \right]}_{+}, \end{array}$$

where *f*(*x*_*i*_^*a*^), *f*(*x*_*i*_^*p*^), and *f*(*x*_*i*_^*n*^) are the corresponding feature expression obtained by training a parameter in the triplet; *α* represents the minimum interval between the anchor-to-positive distance and the anchor-to-negative distance; the value of [·]_+_ defines the degree of loss.

### 2.4. QL-LSTM Model

Previous analysis shows that LSTM with traditional squared-error loss function could not accurately discover the special relationship between *T*_*s*_ and surrounding predictors. To address this problem, inspired by the study of triplet loss, we combined a predictive model with distance metric learning between the sample features. As far as we know, the method based on distance metric learning between the sample features has not been used to estimate *T*_*s*_ ever. It must be noted that the distance metric learning between the sample features is first proposed in the field of image processing. However, there is no description of the similarity of samples for *T*_*s*_ estimation. In this paper, the clustering method is used to label samples; thus, distance metric learning between the sample features could be further applied in *T*_*s*_ estimation.

**Figure 5:**
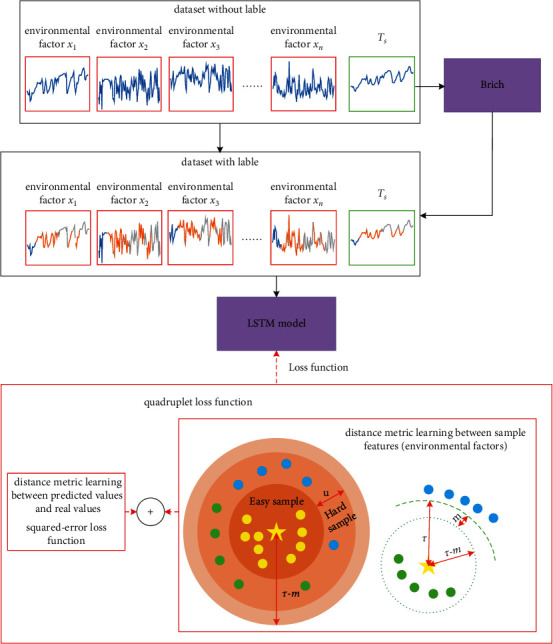
The framework of QL-LSTM.

The framework of our QL-LSTM is shown in Figure 5. Firstly, for its ability to cluster data efficiently and scalability, the *T*_*s*_ data were quantized by the clustering method (called Birch) [34]. In the quantization step, any *T*_*s*_ data quantized to the same label will be defined as similar samples (positive). In contrast, any *T*_*s*_ data quantized to different labels will be defined as the dissimilar samples (negative). It is worthwhile to observe that the number of labels should be neither too large nor too small [35]. Hence, the Calinski Harabasz Score (*CH*) and Y_Silhouette_score (*S*) are used to evaluate the quality of the cluster [36]. The larger value of CH or *S*, the better quality of the clustering results. Second, the labeled data are input into the predictive model (LSTM network). Finally, the weights of the predictive model are updated to reduce the loss based on our quadruplet loss function.

We set *X*={(*x*_*i*_, *l*_*i*_)}_*i*=1_^*N*^ as the input data, where *l*_*i*_ represents *T*_*s*_ labeled as “*i*” and *x*_*i*_ represents the labeled environmental factors. Assume C is the total number of labels, where *l*_*i*_ ∈ [1,2,3 …, *C*]. Then, we project an instance *x*_*i*_ onto the estimate *T*_*s*_ by *f*_LSTM_(.; *θ*) : *R*^*d*^⟶*S*^1^, where *f*_LSTM_ is an LSTM network parameterized by *θ*. Let {*X*_*i*_^*c*^}_*i*=1_^*N*_*c*_^ be the environmental factors in the *i* -th labeled samples. *N*_*c*_ represents the total number of samples. We evaluate the similarity between samples through cluster analysis and expect the output of the model closer to the true value.

#### 2.4.1. Hard Sample Mining

Hard sample mining generally refers to hard negative mining. Adding negative sample sets to participate in model training can improve the effectiveness of learning and training and mine hard negatives as much as possible [37, 38]. For each fixed picture, the farthest sample picture and the nearest negative sample picture in a training batch are applied to train the network to enhance the generalization ability of the network, so that the network can learn better representations.

Inspired by TriHard loss, we first define *x*_*i*_^*c*^ as the test sample: *P*_*c*,*i*_(*P*_*c*,*i*_={*x*_*j*_^*c*^*|j* ≠ *i*}, |*P*_*c*,*i*_|=*N*_*c*_ − 1) is a collection, which includes the samples with the same label; *N*_*c*,*i*_(*N*_*c*,*i*_={*x*_*j*_^*k*^*|k* ≠ *c*}, |*N*_*c*,*i*_|=∑_*k*≠*c*_*N*_*k*_) represents the other samples' collection. (*x*_*i*_^*c*^, *y*_*i*_^*c*^, *P*_*c*,*i*_^*∗*^, *N*_*c*,*i*_^*∗*^) is the quadruplet data set we defined. *P*_*c*,*i*_^*∗*^ is the positive set; *N*_*c*,*i*_^*∗*^ represents the negative set, |*P*_*c*,*i*_^*∗*^| and |*N*_*c*,*i*_^*∗*^| represent positive and negative sample pairs, and these tuples form the training sample pairs. The query sample is represented as *x*_*i*_^*c*^; when *S*_*ij*_^+^ satisfies the formula (3), {*x*_*i*_, *x*_*j*_} is the pair that we need.

(3) $$\begin{array}{c} S_{ij}^{+}>\underset{x_{k}\in P_{c,i}}{\min}S_{ik}+\mu , \end{array}$$

where *S*_*ij*_^+^〈*f*_LSTM_(*x*_*i*_; *θ*), *f*_LSTM_(*x*_*j*_; *θ*)〉 represents the similarity between two samples, where 〈·, ·〉 represents the calculation of an *n* × *n* similarity matrix. *S*_*ij*_ is the element in *S* at (*x*_*i*_, *x*_*j*_), and *μ* as a hyperparameter impacts the quadruplet that can control the number of hard positive samples. The condition for selecting a hard and negative pair is the same as above:

(4) $$\begin{array}{c} S_{ij}^{-}<\underset{x_{k}\in N_{c,i}}{\max}S_{ik}-\mu . \end{array}$$

#### 2.4.2. Optimization Objective

For each test sample *x*_*i*_^*c*^, we use the margin *m* to make it as close to the positive set *P*_*c*,*i*_ as possible, and as far away from the negative set *N*_*c*,*i*_ as possible. All the nontrivial positive points in *P*_*c*,*i*_ are pulled together by minimizing:

(5) $$\begin{array}{c} L_{p}\left(x_{i}^{c};f_{\mathrm{LSTM}}\right)=\frac{1}{2}\sum_{x_{i}^{c}\in P_{c,i}^{*}}{\left({\left[\left\|f_{\mathrm{LSTM}}\left(x_{i}^{c}\right)-f_{\mathrm{LSTM}}\left(x_{j}^{c}\right)\right\|-\left(r-m\right)\right]}_{+}\right)}^{2}, \end{array}$$

where *f*_*LSTM*_(*x*_*i*_^*c*^) and *f*_*LSTM*_(*x*_*j*_^*c*^) denote the estimated *T*_*s*_ of samples *x*_*i*_^*c*^ and *x*_*j*_^*c*^, respectively, and ‖*f*_*LSTM*_(*x*_*i*_^*c*^) − *f*_*LSTM*_(*x*_*j*_^*c*^)‖ is the Euclidean distance between *f*_*LSTM*_(*x*_*i*_^*c*^) and *f*_*LSTM*_(*x*_*j*_^*c*^). Similarly, all nontrivial negative points in *N*_*c*,*i*_ need to push out of the boundary *τ*, by minimizing:

(6) $$\begin{array}{c} L_{N}\left(x_{i}^{c};f_{\mathrm{LSTM}}\right)=\frac{1}{2}\sum_{x_{j}^{k}\in N_{c,i}^{*}}{\left({\left[\tau -\left\|f_{\mathrm{LSTM}}\left(x_{i}^{c}\right)-f_{\mathrm{LSTM}}\left(x_{j}^{k}\right)\right\|\right]}_{+}\right)}^{2}. \end{array}$$

Meanwhile, we applied the squared-error loss function to the LSTM model for *T*_*s*_ estimation, as follows:

(7) $$\begin{array}{c} L_{MSE}\left(x_{i}^{c};f_{\mathrm{LSTM}}\right)=\frac{1}{2}\sum_{x_{i}^{c}\in N_{c}}{\left(f_{\mathrm{LSTM}}\left(x_{i}^{c}\right)-y_{i}^{c}\right)}^{2}. \end{array}$$

In the QL-LSTM, three minimization objectives were put into the model, and they are optimized at the same time:

(8) $$\begin{array}{c} L_{\mathrm{QL-LSTM}}\left(x_{i}^{c};f_{\mathrm{LSTM}}\right)=L_{P}\left(x_{i}^{c};f_{\mathrm{LSTM}}\right)+L_{N}\left(x_{i}^{c};f_{\mathrm{LSTM}}\right)+L_{\mathrm{MSE}}\left(x_{i}^{c};f_{\mathrm{LSTM}}\right), \end{array}$$

We incorporate stochastic gradient descent and minibatch into the QL-LSTM to optimize the estimation model.

**Figure 6:**
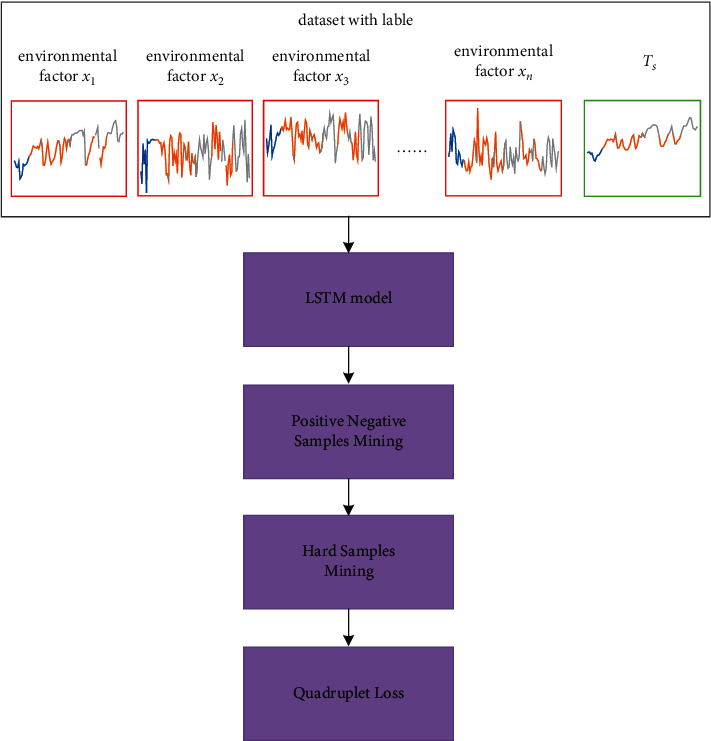
The learning procedure of QL-LSTM.

*x*_*i*_^*c*^ is a sample of the minibatch, which is obtained by sampling the labels of the training samples randomly, and serves as an anchor. We represent the QL-LSTM of each minibatch as

(9) $$\begin{array}{c} L_{QL-LSTM}\left(X;f\right)=\frac{1}{N}\sum_{\forall_{c}\forall_{i}}L_{QL-LSTM}\left(x_{i}^{c};f\right), \end{array}$$

where *N* denotes the batch size.  Figure 6 represented the learning procedure of our QL-LSTM model.

### 2.5. Model Training and Testing

The input of our model is the corresponding meteorological data (*T*_*a*_, *W*, *A*_*p*_, *R*_*s*_, *VPD,* and *T*_*s*_) from Laegern and Fluehli stations in Switzerland. And we downloaded the data at https://fluxnet.fluxdata.org/ on FLUXNET with a total of 3,287 patterns from 2006 to 2014. Training datasets had 2465 patterns, and the rest as testing datasets.

Comparing our QL-LSTM model with the other advanced methods (SVR, BPNN, ELM, and LSTM), meanwhile, we calculate several evaluation criteria to analyze the model performance, including model fitting degree and the accuracy of the estimation model, as follows:

(10) $$\begin{array}{c} \mathrm{RMSE}=\sqrt{\frac{\sum_{n=1}^{N}{\left(y_{i}-{\hat{y}}_{i}\right)}^{2}}{N},}\\ \mathrm{WI}=1-\frac{\sum_{n=1}^{N}{\left(y_{i}-{\hat{y}}_{i}\right)}^{2}}{\sum_{n=1}^{N}{\left(\left|{\hat{y}}_{i}-\bar{y}\right|+\left|y_{i}-\bar{y}\right|\right)}^{2}},\\ \mathrm{NS}=1-\frac{\sum_{n=1}^{N}{\left(y_{i}-{\hat{y}}_{i}\right)}^{2}}{\sum_{n=1}^{N}{\left(y_{i}-\bar{y}\right)}^{2}},\\ \mathrm{MAE}=\frac{\sum_{n=1}^{N}\left|y_{i}-{\hat{y}}_{i}\right|}{N},\\ \mathrm{LMI}=1-\frac{\sum_{n=1}^{N}\left|y_{i}-{\hat{y}}_{i}\right|}{\sum_{n=1}^{N}\left|y_{i}-\bar{y}\right|}, \end{array}$$

where *N* is the number of the whole data, *y*_*i*_ denotes the observed value, ${\hat{y}}_{i}$ is the predicted value, and $\bar{y}$ is the average of the true values.

### 2.6. Experiments

The data within half an hour is obtained from two meteorological stations in an ecological nature reserve, located in Switzerland, namely, Legern and Fluley. The corresponding meteorological data (*T*_*a*_, *W*, *A*_*p*_, *R*_*s*_, *VPD,* and *T*_*s*_) and past *T*_*s*_ data were input into the models. Meanwhile, the input variables are normalized to eliminate the dimensional influence between indicators. And the formula is as follows:

(11) $$\begin{array}{c} x_{\text{norm}}=\frac{x-x_{\min}}{x_{\max}-x_{\min}}, \end{array}$$

where the minimum value of the sample data is represented by *x*_min_, and the maximum value is represented by *x*_max_. Moreover, we have conducted research on the influence of the surrounding environmental factors on the model prediction. And we found the value of *R*_*s*_ in the data of the two stations is low, which is close to the normal distribution.

We conducted a statistical analysis of the data from the two stations.  Table 1 listed the details of variables (minimum value (*x*_min_), maximum value (*x*_max_), average value (*x*_mean_), standard deviation (*z*_*sd*_), skewness (*z*_*s*_), and variation coefficient (*z*_*v*_)). We used the daily data to verify the performance of the model with every half an hour data. The results showed in Table 1 that *A*_*p*_ had the highest negative skewness and presents a normal distribution at 5 cm depth, which presented similar characteristics in both stations. Meanwhile, *z*_*v*_ showed the biggest difference between the two stations. *T*_*s*_ at the 5 cm, 10 cm, and 15 cm depths range −1.888–26.876°C, −0.181–22.193°C, and 0.16–20.826°C, respectively. In summary, results showed that the values of *z*_*sd*_, *z*_*s*_, and *z*_*v*_ change very slightly.

## 3. Results and Discussion

For testing the superiority of our QL-LSTM model performance for *T*_*s*_ estimation using scikit-learn, we compared our test results with those of other advanced models (SVR, BPNN, ELM, and LSTM).

We choose default parameters for the SVR model. For the BPNN model, the square error is used as the loss function, and the optimization is Adam. The number of samples selected for the model is 400, the iteration is set to 500, the learning rate is set to 5.0e-4, and the size of the nodes is set to 128. The elm function was used to model the ELM model, the sigmoid served to activate the function in the hidden layer, and we set the same size of the nodes to BPNN. Furthermore, we set the hyperparameters of the LSTM to be the same as that of QL-LSTM. As can be seen from Table 2 and 3, the different values of the hyperparameter can generate the different predictive results. When the number of samples selected for the model is set to 400, the iteration to 500, the num_QL−LSTM_ to 128, and set the learning rate to 1.0e-3, the QL-LSTM model has the best performance.

### 3.1. Evaluation for the Hyperparameters in Quadruplet Loss Function

**Figure 7:**
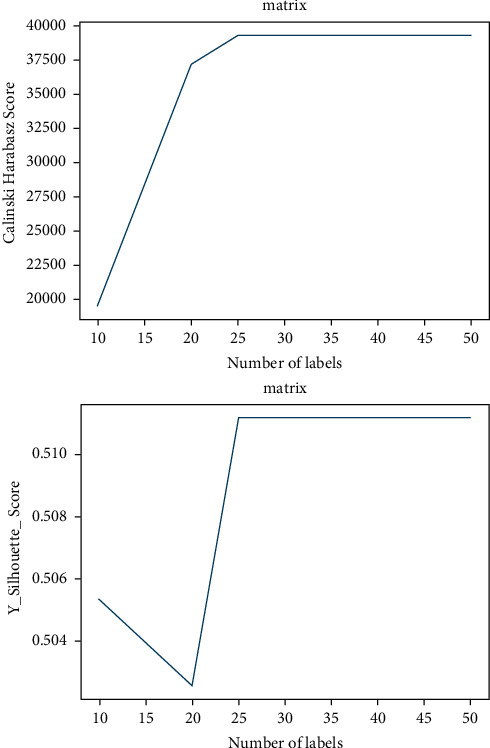
The Calinski Harabasz Score and Y_Silhouette_score with different numbers of labels.

**Figure 8:**
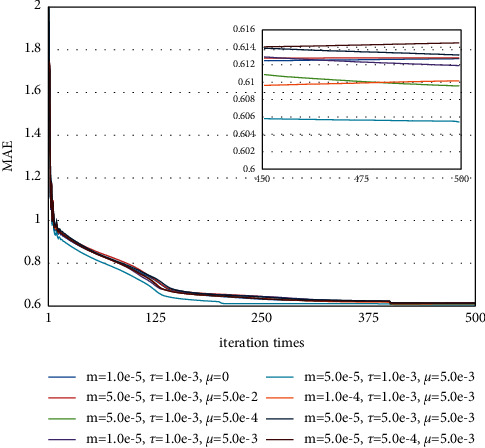
The estimation results with different *μ*, *τ,* and *m* at Laegern meteorological station.

The quadruplet loss function has five main hyperparameters, which are the total number of labels *C*, hyperparameter *μ* in equations (3) and (4), and *τ* and *m* in equations (5) and (6). When we evaluate the above hyperparameters in the quadruplet loss function, we set the parameters num_*QL*−LSTM_ to 128, the learning rate to 1.0e-3, the iteration time to 500, and the batch size to 400. We first select the best *C* based on the Calinski Harabasz Score and Y_Silhouette_score.  Figure 7 denotes the Calinski Harabasz Score and Y_Silhouette_score with different numbers of labels. It is observed that both scores achieve the best result when *C* is 25. Then, we gradually tune the hyperparameters, *τ* and *m*.  Figure 8 represented the results of the estimation model with different *μ*, *τ*, and *m* in Laegern meteorological station. We can see that when we set *μ* to be 5.0e-3, *τ* to be 1.0e-3, and *m* to be 5.0e-5, and our QL-LSTM model could achieve the best estimation performance (RMSE = 0.789, MAE = 0.605, NS = 0.977, WI = 0.994, and LMI = 0.865). It is probably because the smaller hyperparameters we set, the less hard samples would be computed. Meanwhile, when we set the larger hyperparameters, the more redundant samples would be computed.

### 3.2. The Impact of Different Inputs on the Performance of the Predictive Model

In this part, we analyzed the environmental factors that may affect our QL-LSTM model for *T*_*s*_ estimation. Considering that the interaction between different environmental factors would have an impact on the *T*_*s*_ estimation, we combine the meteorological variables accordingly and input them into the submodels we set as follows:

- Input *I*_*1*_: *T*_*a*_ (d − 1)
- Input *I*_*2*_: *T*_*a*_ (d − 1) + *R*_*s*_(d − 1)
- Input *I*_*3*_: *T*_*a*_ (d − 1) + *R*_*s*_(d − 1) + *VPD*(d − 1)
- Input *I*_*4*_: *T*_*a*_ (d − 1) + *R*_*s*_(d − 1) + *VPD*(d − 1) + *W*(d − 1)
- Input *I*_*5*_: *T*_*a*_ (d − 1) + *R*_*s*_(d − 1) + *VPD*(d − 1) + *W*(d − 1) + *A*_*p*_(d − 1)
- Output: *T*_*s*_ (d)

Then, we consider that the past *T*_*s*_ will continue to have an impact on the future *T*_*s*_ estimation, so we have carried out lag processing for the past *T*_*s*_ on different days, as follows:

- Input *I*_*6*_: *T*_*s*_ (d − 1)
- Input *I*_*7*_: *T*_*s*_ (d − 1) + *T*_*s*_ (d − 2)
- Input *I*_*8*_: *T*_*s*_ (d − 1) + *T*_*s*_ (d − 2) + *T*_*s*_ (d − 3)
- Input *I*_*9*_: *T*_*s*_ (d − 1) + *T*_*s*_ (d − 2) +*T*_*s*_ (d − 3) + *T*_*s*_ (d − 4)
- Input *I*_*10*_: *T*_*s*_ (d − 1) + *T*_*s*_ (d − 2) +*T*_*s*_ (d − 3) + *T*_*s*_ (d − 4) + *T*_*s*_ (d − 5)
- Output: *T*_*s*_ (d)

We input what we specified above into QL-LSTM to predict the *T*_*s*_ (d) at the 5 cm depth of the Laegern station. For our model, we first selected the hyperparameters *μ* as 5.0e-3, *τ* as 1.0e-3, *m* as 5.0e-5, *C* as 25, num_QL−LSTM_ as 128, learning rate as 1.0e-3, iteration time as 500, and batch size as 400, and the results are presented in Table 4. Obviously, the methods of QL-LSTM(*I*_*3*_) and QL-LSTM(*I*_*8*_) are better than the others, respectively. Meanwhile, we could conclude that *W* (d − 1), *A*_*p*_ (d − 1), *T*_*s*_ (d − 4), and *T*_*s*_ (d − 5) all have an influence on the performance of the predictive model. In addition, by comparing the estimation results between meteorological variables input and past *T*_*s*_ input, we found that our model with past *T*_*s*_ could achieve greater accuracy in modeling than the one with meteorological variables. The reason may be that the predictive model with past *T*_*s*_ input has stronger memory for *T*_*s*_ variables. The estimation of the future *T*_*s*_ should make the best use of its continuity; in this way, we can make a reliable *T*_*s*_ estimation, which not only continues its historical tendency but also conforms to its actual performance. Hence, we construct the predictive model (QL-LSTM(I_11_)) by combining the environmental factors (*T*_*a*_ (d − 1), *R*_*s*_(d − 1), *VPD*(d − 1)) with past *T*_*s*_ (*T*_*s*_ (d − 1), *T*_*s*_ (d − 2), *T*_*s*_ (d − 3)), which is also considered in estimating the *T*_*s*_ (d) at the 5 cm depth of the Laegern station. Experiment results prove that it could achieve the best estimation performance (RMSE = 0.789, LMI = 0.865, WI = 0.994, NS = 0.977, and MAE = 0.605). Hence, the final input for the predictive models is the environmental factors (*T*_*a*_(d − 1), *R*_*s*_(d − 1), *VPD*(d − 1)) and the past *T*_*s*_ (*T*_*s*_ (d − 1), *T*_*s*_ (d − 2), *T*_*s*_ (d − 3)).

**Figure 9:**
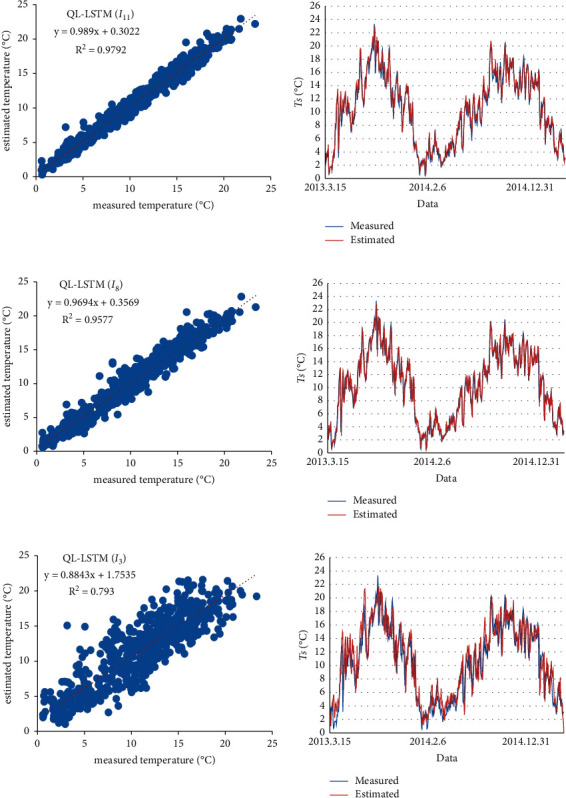
The scatterplots of the predictive model testing results (the values of estimated and observed) for the Laegern station. (a) QL-LSTM(I11), (b) QL-LSTM(I8) model, and (c) QL-LSTM(I3) model.

**Figure 10:**
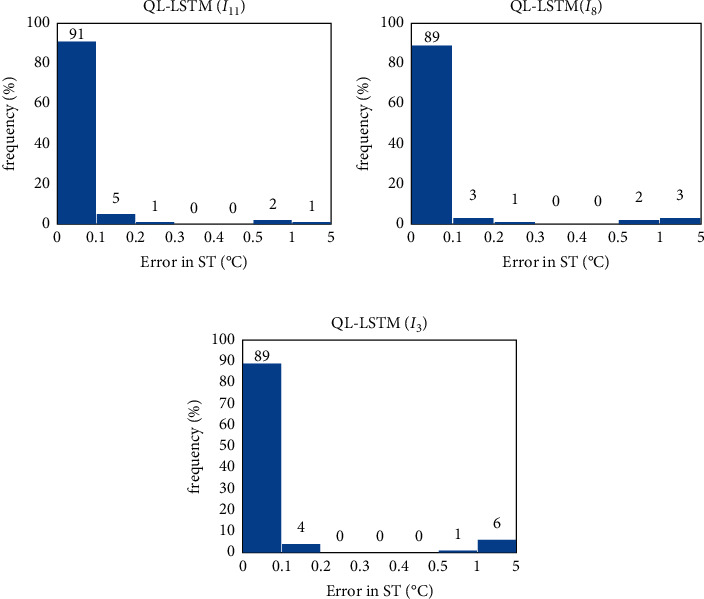
The frequency plot of the predictive models (absolute estimation error) for the Laegern station. (a) QL-LSTM(I11), (b) QL-LSTM(I8) model, and (c) QL-LSTM(I3) model.

The three methods (QL-LSTM(I_3_), QL-LSTM(I_8_), and QL-LSTM(I_11_)) are used to test the data of the Laegern station.  Figure 9 shows the linear relationship between the predicted value and the observed value. The QL-LSTM(I_11_) model gets the best predictive performance with *y* = 0.9899*x* + 0.3022 and the higher *R*^2^ (0.9792) compared with the others. In the frequency diagram (Figure 10) of the models (QL-LSTM(I_3_), QL-LSTM(I_8_), and QL-LSTM(I_11_)), the QL-LSTM(I_11_) also has a higher frequency (91%) compared to the others. Therefore, we can draw a conclusion that the predictive model (a combination of the environmental factors and the past *T*_*s*_) normally outperformed the other two (by either past measurements of *T*_*s*_ or other meteorological information) in the *T*_*s*_ estimation.

### 3.3. Comparison with Different Models

In this part, our QL-LSTM model was compared with several advanced models, including SVR, BPNN, ELM, and LSTM. The data of *T*_*a*_, *R*_*s*_, and *VPD* on day “d − 1”, and *T*_*s*_ data on different days were acted as input data to different predictive models, and the output was the predicted value of *T*_*s*_ on days “d”, “d + 5”, and “d + 15”. Time steps were in days.

The testing results of five different models at 5, 10, and 15 cm depth on the 1st, 5th, and 15th days of the Laegern station were shown in Table 5. And we can see that our QL-LSTM model performs better than the existing advanced models at the 5 cm depth on the 1st day. Specifically, the value of RMSE is 0.789, which is reduced relative to 13% (LSTM), 22% (ELM), 28% (BPNN), and 22% (SVR), respectively. The MAE values amount to 0.605 (QL-LSTM), and the others are 0.813 (SVR), 0.872 (BPNN), 0.824 (ELM), and 0.821 (LSTM). Meanwhile, the QL-LSTM model achieved a higher value of NS, WI, and LMI. Hence, it is obvious that our model had the best performance in this case. For the results of 5 cm depth on the 15th day, the LSTM achieved a higher WI (0.892) than estimation from other models on the 15th day, but it is similar to the WI (0.891) of our model. For 10 and 15 cm depth results on the 1st, 5^th^, and 15th days, the performance of our QL-LSTM model remains stable, although our QL-LSTM model has the lower values of WI (0.952 and 0.933) than the values of WI (0.954 and 0.934) on the LSTM model in individual cases. It can be found that the predictive performance will get better as the soil depth decreases (from 5 cm to 15 cm), but it will decrease as time goes on (from 1st to 15th days). The systematic errors caused this phenomenon for long-term estimation [39].

The same strategy was applied in the Fluehli station to further verify the performance of the models, with the results shown in Table 6. And our QL-LSTM model performs better compared with others. However, for 5 cm depth on the 15th day and 15 cm depth on the 5th day, the BPNN model performs better with the results of RMSE = 2.081, LMI = 0.584, WI = 0.937, NS = 0.769, and MAE = 2.099, and RMSE = 1.832, LMI = 0.726, WI = 0.973, NS = 0.897, and MAE = 1.352 and LMI = 0.726. Our method does not perform well in some cases probably because the weights of the LSTM model are randomly selected to generate the nonoptimal solution. Meanwhile, our novel loss function (quadruplet loss) is applied based on the LSTM model; it only improved estimation performance to a certain extent against the LSTM model. All in all, the results of our testing on the data of different regions show that the performance of our QL-LSTM model is usually better for *T*_*s*_ prediction with different depths and days.

## 4. Conclusions

Soil temperature (*T*_*s*_) is a main physical variable of the land surface, which has an impact on many aspects, such as the growth and yield of crops. Therefore, how to predict *T*_*s*_ accurately is very important. This paper proposed the QL-LSTM model and compared it with the state-of-the-art predictive models to use the meteorological data and past *T*_*s*_ of the Laegern and Fluehli stations (Switzerland) for daily *T*_*s*_ estimation at 5, 10, and 15 cm depth on the 1st, 5th, and 15th days. The experiment results showed that the QL-LSTM model performed better than the existing advanced models for *T*_*s*_ estimation in multifarious cases.

In addition, to enrich processing information in loss function and further improve estimation performance, we attempt to design the novel quadruplet loss function that combines the traditional squared-error loss function with distance metric learning between the sample features. Similar samples can be zoomed and the dissimilar samples can be pushed.

The distance metric learning between the sample features is combined with the squared-error loss function, which could improve the estimation performance to a certain extent. However, the many hyperparameters in our method may cause sensitivity issues in estimation, which may lead to poor generalization ability of other estimations. In the future, the parametric adaptive method will be explored for a new loss function in the follow-up study.

## Figures and Tables

**Table 1:** Statistical results of the applied data for Laegern and Fluehli stations.

Station	Variable	*x*_min_	*x*_max_	*x*_mean_	*z*_*s* *d*_	*z*_*s*_	*z*_*v*_
Laegern	*T*_*a*_ (°C)	−14.509	23.646	7.857	7.084	−0.118	0.901
	*R*_*s*_ (W/m^2^)	175.545	379.458	305.081	35.164	−0.418	0.115
	VPD (hpa)	0.54	15.937	3.271	2.334	1.424	0.713
	W (m/s)	0.668	8.025	2.237	1.005	1.411	0.449
	*A*_*p*_ (kpa)	89.876	95.163	93.237	0.714	-0.630	0.007
	*T*_*s*_ −5 cm (°C)	−1.888	26.876	10.104	6.061	0.103	0.599
	*T*_*s*_ −10 cm (°C)	−0.181	22.193	9.726	5.435	−0.031	0.558
	*T*_*s*_ −15 cm (°C)	0.16	19.394	9.010	5.025	−0.068	0.557

Fluehli	*T*_*a*_ (°C)	−14.448	22.877	7.708	6.906	−0.100	0.895
	*R*_*s*_ (W/m^2^)	194.734	377.444	306.015	32.805	−0.327	0.107
	VPD (hpa)	0.416	9.662	2.129	1.543	1.410	0.724
	W (m/s)	0.342	4.636	1.476	0.619	0.894	0.419
	*A*_*p*_ (kpa)	82.308	87.164	85.493	0.711	−0.843	0.008
	*T*_*s*_ −5 cm (°C)	−0.35	21.822	8.729	6.338	0.075	0.726
	*T*_*s*_ −10 cm (°C)	−0.044	21.727	8.836	6.242	0.071	0.706
	*T*_*s*_ −15 cm (°C)	0.432	20.826	8.813	6.023	0.062	0.683

**Table 2:** Predictive performance with different num_QL−LSTM_ and learning rates at Laegern station.

Learning rate	num_*QL*−LSTM_	RMSE	MAE	NS	WI	LMI
1.0e-5	16	4.735	4.059	0.1799	0.325	0.093
	32	2.970	2.508	0.677	0.845	0.439
	64	1.346	1.061	0.933	0.980	0.763
	128	1.365	1.073	0.931	0.982	0.760
	256	1.341	1.049	0.934	0.983	0.765
	16	1.230	0.962	0.944	0.986	0.785

1.0e-4	32	1.175	0.915	0.949	0.987	0.795
	64	1.167	0.903	0.950	0.987	0.798
	128	1.159	0.884	0.950	0.987	0.802
	256	1.148	0.871	0.951	0.987	0.805
	16	1.129	0.855	0.953	0.988	0.808

5.0e-4	32	1.076	0.809	0.957	0.989	0.819
	64	1.001	0.757	0.963	0.990	0.830
	128	0.817	0.629	0.975	0.993	0.859
	256	0.852	0.657	0.973	0.993	0.853
	16	1.069	0.802	0.958	0.989	0.820

1.0e-3	32	0.950	0.716	0.966	0.991	0.839
	64	0.880	0.676	0.971	0.992	0.848
	**128**	**0.817**	**0.625**	**0.975**	**0.993**	**0.860**
	256	0.825	0.634	0.975	0.993	0.858
	16	0.871	0.675	0.972	0.993	0.849

5.0e-3	32	0.858	0.661	0.973	0.993	0.852
	64	0.868	0.661	0.972	0.993	0.852
	128	0.852	0.648	0.973	0.993	0.855
	256	0.854	0.649	0.973	0.993	0.855

**Table 3:** Predictive performance with different numbers of batch size and iterations at Laegern station.

Batch	Iteration	RMSE	MAE	NS	WI	LMI
100	100	0.860	0.666	0.972	0.993	0.851
	200	0.873	0.676	0.972	0.993	0.848
	500	0.880	0.667	0.971	0.992	0.850
	800	1.073	0.825	0.957	0.989	0.815
	100	0.849	0.654	0.973	0.993	0.853

200	200	0.817	0.625	0.975	0.993	0.860
	500	0.843	0.642	0.973	0.993	0.856
	800	0.916	0.692	0.969	0.992	0.845
	100	0.973	0.739	0.965	0.991	0.834

300	200	0.831	0.644	0.974	0.993	0.856
	500	0.817	0.626	0.975	0.993	0.860
	800	0.869	0.664	0.972	0.993	0.851
	100	1.038	0.784	0.960	0.990	0.824

400	200	0.851	0.654	0.973	0.993	0.853
	**500**	**0.809**	**0.622**	**0.976**	**0.993**	**0.860**
	800	0.817	0.628	0.975	0.993	0.859
	100	1.076	0.812	0.957	0.989	0.818

500	200	0.868	0.666	0.972	0.993	0.851
	500	0.809	0.623	0.976	0.993	0.860
	800	0.811	0.626	0.975	0.993	0.860

**Table 4:** Predictive performance of QL-LSTM at 5 cm depth for the Laegern station.

Method	RMSE	MAE	NS	WI	LMI
QL-LSTM(_I1_)	1.469	1.143	0.921	0.980	0.744
QL-LSTM(_I2_)	1.444	1.113	0.923	0.981	0.751
QL-LSTM(_I3_)	**1.221**	**0.937**	**0.945**	**0.986**	**0.790**
QL-LSTM(_I4_)	1.396	1.078	0.928	0.982	0.759
QL-LSTM(_I5_)	1.454	1.143	0.922	0.980	0.744
QL-LSTM(_I6_)	1.143	0.866	0.952	0.987	0.806
QL-LSTM(_I7_)	1.095	0.823	0.956	0.988	0.815
QL-LSTM(_I8_)	**1.077**	**0.815**	**0.957**	**0.989**	**0.817**
QL-LSTM(_I9_)	1.096	0.834	0.956	0.988	0.813
QL-LSTM(_I10_)	1.115	0.842	0.954	0.988	0.811

**Table 5:** The predictive performance with different models at the Laegern station.

Depth (cm)	Day	Method	RMSE	MAE	NS	WI	LMI
5	d	SVR	1.007	0.813	−1315.5	0.412	−882.6
		BPNN	1.101	0.872	0.930	0.982	0.838
		ELM	1.016	0.824	0.925	0.983	0.849
		LSTM	0.910	0.821	0.926	0.983	0.850
		QL-LSTM	**0.789**	**0.605**	**0.977**	**0.994**	**0.865**
	d + 5	SVR	2.665	2.133	-1205.3	0.419	-845.1
		BPNN	2.812	2.230	0.700	0.919	0.535
		ELM	2.832	2.261	0.693	0.916	0.528
		LSTM	2.643	2.113	0.730	0.926	0.560
		QL-LSTM	**2.436**	**1.908**	**0.782**	**0.937**	**0.573**
	d + 15	SVR	3.322	2.680	−1215.2	0.422	−846.9
		BPNN	3.271	2.657	0.601	0.886	0.438
		ELM	3.403	2.723	0.572	0.881	0.424
		LSTM	3.278	2.652	0.601	**0.892**	0.439
		QL-LSTM	**3.078**	**2.428**	**0.651**	0.891	**0.455**

10	d	SVR	1.011	0.850	−1291.5	0.414	−870.2
		BPNN	0.986	0.831	0.923	0.983	0.837
		ELM	1.090	0.862	0.916	0.981	0.830
		LSTM	0.980	0.824	0.923	0.983	0.839
		QL-LSTM	**0.761**	**0.605**	**0.975**	**0.993**	**0.854**
	d + 5	SVR	2.173	1.752	−1211.9	0.417	−842.1
		BPNN	2.188	1.751	0.774	0.935	0.608
		ELM	2.291	1.833	0.762	0.938	0.596
		LSTM	2.181	1.745	0.781	**0.954**	0.615
		QL-LSTM	**1.973**	**1.545**	**0.833**	0.952	**0.628**
	d + 15	SVR	2.794	2.220	−1244.5	0.431	−850.8
		BPNN	2.831	2.263	0.652	0.909	0.493
		ELM	2.920	2.300	0.632	0.899	0.484
		LSTM	2.763	2.131	0.667	0.907	0.509
		QL-LSTM	**2.538**	**1.975**	**0.724**	**0.917**	**0.524**

15	d	SVR	0.531	0.450	−1304.5	0.424	−873.5
		BPNN	0.525	0.442	0.942	0.987	0.921
		ELM	0.637	0.488	0.940	0.987	0.913
		LSTM	0.512	0.436	0.944	0.988	0.927
		QL-LSTM	**0.312**	**0.239**	**0.995**	**0.998**	**0.938**
	d + 5	SVR	1.761	1.400	−1262.3	0.426	−857.9
		BPNN	1.773	1.441	0.826	0.957	0.667
		ELM	1.921	1.532	0.800	0.950	0.643
		LSTM	1.742	1.408	0.831	0.958	0.675
		QL-LSTM	**1.533**	**1.203**	**0.882**	**0.968**	**0.687**
	d + 15	SVR	2.396	1.937	−1266.3	0.431	−857.4
		BPNN	2.406	1.926	0.707	0.933	0.537
		ELM	2.508	2.008	0.674	0.925	0.518
		LSTM	2.401	1.918	0.707	**0.934**	0.539
		QL-LSTM	**2.189**	**1.719**	**0.760**	**0.933**	**0.553**

**Table 6:** The predictive performance with different models at the Fluehli station.

Depth (cm)	Day	Method	RMSE	MAE	NS	WI	LMI
5	d	SVR	0.691	0.549	−1311.5	0.429	−868.5
		BPNN	0.718	0.550	0.942	0.918	0.916
		ELM	0.693	0.538	0.942	0.988	0.918
		LSTM	0.723	0.549	0.941	0.987	0.916
		QL-LSTM	**0.492**	**0.352**	**0.992**	**0.998**	**0.930**
	d + 5	SVR	2.316	1.745	−1266.6	0.431	−851.7
		BPNN	2.297	1.716	0.820	0.955	0.687
		ELM	2.463	1.880	0.799	0.949	0.654
		LSTM	2.291	1.718	0.821	0.956	0.686
		**QL-LSTM**	**2.084**	**1.526**	**0.872**	**0.966**	**0.698**
	d + 15	SVR	3.023	2.316	−1257.5	0.433	−847.4
		BPNN	**2.801**	**2.099**	**0.769**	**0.937**	**0.584**
		ELM	3.143	2.400	0.694	0.918	0.534
		LSTM	2.971	2.353	0.723	0.922	0.557
		QL-LSTM	2.815	2.165	0.766	0.933	0.570

10	d	SVR	0.628	0.518	−1310.1	0.428	−870.4
		BPNN	0.635	0.508	0.944	0.988	0.926
		ELM	0.621	0.510	0.944	0.988	0.926
		LSTM	0.610	0.500	0.944	0.988	0.928
		QL-LSTM	**0.381**	**0.286**	**0.995**	**0.998**	**0.941**
	d + 5	SVR	2.043	1.529	−1274.5	0.430	−856.9
		BPNN	2.021	1.510	0.846	0.963	0.721
		ELM	2.153	1.600	0.830	0.958	0.697
		LSTM	**1.777**	1.506	**0.901**	**0.973**	**0.732**
		**QL-LSTM**	1.794	**1.307**	0.899	0.973	0.732
	d + 15	SVR	2.813	2.143	−1271.7	0.432	−854.43
		BPNN	2.792	2.112	0.738	0.933	0.594
		ELM	2.926	2.300	0.717	0.926	0.558
		LSTM	2.802	2.108	0.736	0.932	0.597
		QL-LSTM	**2.601**	**1.906**	**0.789**	**0.948**	**0.612**

15	d	SVR	0.631	0.506	−1291.9	0.430	−862.9
		BPNN	0.642	0.500	0.943	0.988	0.926
		ELM	**0.407**	0.468	0.944	0.988	0.930
		LSTM	0.618	0.465	0.944	0.988	0.931
		QL-LSTM	0.409	**0.290**	**0.994**	**0.998**	**0.941**
	d + 5	SVR	2.028	1.551	−1269.3	0.429	−853.7
		BPNN	**1.832**	**1.352**	**0.897**	**0.973**	**0.726**
		ELM	2.173	1.638	0.830	0.958	0.691
		LSTM	2.042	1.555	0.846	0.962	0.715
		QL-LSTM	1.835	1.354	0.896	0.972	0.725
	d + 15	SVR	2.771	2.156	−1257.7	0.433	−848.4
		BPNN	2.762	2.134	0.748	0.935	0.595
		ELM	2.802	2.200	0.741	0.931	0.579
		LSTM	2.795	2.112	0.742	0.933	0.597
		QL-LSTM	**2.552**	**1.931**	**0.805**	**0.949**	**0.608**

## Data Availability

The experimental data are available without any restriction.

## References

[1] Keshavarzi A., Sarmadian F., Omran E. S. E., Iqbal M. (2015). A neural network model for estimating soil phosphorus using terrain analysis. *The Egyptian Journal of Remote Sensing and Space Science*.

[2] Lai L., Zhao X., Jiang L. (2012). Soil respiration in different agricultural and natural ecosystems in an arid region. *PLoS One*.

[3] Tabari H., Hosseinzadeh Talaee P., Willems P. (2014). Short-term forecasting of soil temperature using artificial neural network. *Meteorological Applications*.

[4] Wang W., Akhtar K., Ren G., Yang G., Feng Y., Yuan L. (2019). Impact of straw management on seasonal soil carbon dioxide emissions, soil water content, and temperature in a semi-arid region of China. *The Science of the Total Environment*.

[5] Silva B. d. O., Moitinho M. R., Santos G. A. d. A., Teixeira D. D. B., Fernandes C., La Scala N. (2019). Soil CO2 emission and short-term soil pore class distribution after tillage operations. *Soil and Tillage Research*.

[6] Mehdizadeh S., Behmanesh J., Khalili K. (2017). Evaluating the performance of artificial intelligence methods for estimation of monthly mean soil temperature without using meteorological data. *Environmental Earth Sciences*.

[7] Liang L. L., Riveros-Iregui D. A., Emanuel R. E., McGlynn B. L. (2014). A simple framework to estimate distributed soil temperature from discrete air temperature measurements in data-scarce regions. *Journal of Geophysical Research: Atmospheres*.

[8] Liu Y., Weerts A. H., Clark M. (2012). Advancing data assimilation in operational hydrologic forecasting: progresses, challenges, and emerging opportunities. *Hydrology and Earth System Sciences*.

[9] Kisi O., Tombul M., Kermani M. Z. (2015). Modeling soil temperatures at different depths by using three different neural computing techniques. *Theoretical and Applied Climatology*.

[10] Tabari H., Sabziparvar A.-A., Ahmadi M. (2011). Comparison of artificial neural network and multivariate linear regression methods for estimation of daily soil temperature in an arid region. *Meteorology and Atmospheric Physics*.

[11] Moazenzadeh R., Mohammadi B. (2019). Assessment of bio-inspired metaheuristic optimisation algorithms for estimating soil temperature. *Geoderma*.

[12] Delbari M., Sharifazari S., Mohammadi E. (2019). Modeling daily soil temperature over diverse climate conditions in Iran—a comparison of multiple linear regression and support vector regression techniques. *Theoretical and Applied Climatology*.

[13] Feng Y., Cui N., Hao W., Gao L., Gong D. (2019). Estimation of soil temperature from meteorological data using different machine learning models. *Geoderma*.

[14] Sanikhani H., Deo R. C., Yaseen Z. M., Eray O., Kisi O. (2018). Non-tuned data intelligent model for soil temperature estimation: a new approach. *Geoderma*.

[15] Bilgili M. (2011). The use of artificial neural networks for forecasting the monthly mean soil temperatures in Adana, Turkey. *Turkish Journal of Agriculture and Forestry*.

[16] Kisi O., Sanikhani H., Cobaner M. (2017). Soil temperature modeling at different depths using neuro-fuzzy, neural network, and genetic programming techniques. *Theoretical and Applied Climatology*.

[17] Zeynoddin M., Bonakdari H., Ebtehaj I., Esmaeilbeiki F., Gharabaghi B., Zare Haghi D. (2019). A reliable linear stochastic daily soil temperature forecast model. *Soil and Tillage Research*.

[18] Samadianfard S., Asadi E., Jarhan S. (2018). Wavelet neural networks and gene expression programming models to predict short-term soil temperature at different depths. *Soil and Tillage Research*.

[19] Mehdizadeh S., Fathian F., Safari M. J. S., Khosravi A. (2020). Developing novel hybrid models for estimation of daily soil temperature at various depths. *Soil and Tillage Research*.

[20] Nahvi B., Habibi J., Mohammadi K., Shamshirband S., Al Razgan O. S. (2016). Using self-adaptive evolutionary algorithm to improve the performance of an extreme learning machine for estimating soil temperature. *Computers and Electronics in Agriculture*.

[21] Hochreiter S., Schmidhuber J. (1997). Long short-term memory. *Neural Computation*.

[22] Guo J., Xie Z., Qin Y., Jia L., Wang Y. (2019). Short-term abnormal passenger flow prediction based on the fusion of SVR and LSTM. *IEEE Access*.

[23] Zhang J., Zhu Y., Zhang X., Ye M., Yang J. (2018). Developing a Long Short-Term Memory (LSTM) based model for predicting water table depth in agricultural areas. *Journal of Hydrology*.

[24] Qing X., Niu Y. (2018). Hourly day-ahead solar irradiance prediction using weather forecasts by LSTM. *Energy*.

[25] Li Q., Hao H., Zhao Y. (2020). GANs-LSTM model for soil temperature estimation from meteorological: a new approach. *IEEE Access*.

[26] Wang Y., Gan D., Sun M., Zhang N., Lu Z., Kang C. (2019). Probabilistic individual load forecasting using pinball loss guided LSTM. *Applied Energy*.

[27] Kratzert F., Klotz D., Herrnegger M., Sampson A. K., Hochreiter S., Nearing G. S. (2019). Toward improved predictions in ungauged basins: exploiting the power of machine learning. *Water Resources Research*.

[28] Ekpenyong E. J., Sunday G. (2018). Bayes Estimator of the Shape Parameter of the Burr type XII with generalised squared error and Linex loss functions under some prior distributions. *International Journal of Statistics and Applied Mathematics*.

[29] Han M. (2019). E-Bayesian estimation and its E-MSE under the scaled squared error loss function, for exponential distribution as example. *Communications in Statistics—Simulation and Computation*.

[30] Fan L., Zhao H., Zhao H. (2020). Distribution consistency loss for large-scale remote sensing image retrieval. *Remote Sensing*.

[31] Li Q., Xu R., Zhao H., Xu L., Shan X., Gong P. (2018). Computer-aided diagnosis of mammographic masses using local geometric constraint image retrieval. *Optik*.

[32] Fan L., Zhao H., Zhao H., Liu P., Hu H. (2019). Image retrieval based on learning to rank and multiple loss. *ISPRS International Journal of Geo-Information*.

[33] Hermans A., Beyer L., Leibe B. (2017). In Defense of the triplet loss for person Re-identification. Computer vision and pattern recognition (CVPR). https://arxiv.org/abs/1703.07737.

[34] Zhang T., Ramakrishnan R., Livny M. (1997). A new data clustering algorithm and its applications. *Data Mining and Knowledge Discovery*.

[35] Zhao H., Li Q., Liu P. (2014). Hierarchical geometry verification via maximum entropy saliency in image retrieval. *Entropy*.

[36] Xu R., Xu J., Wunsch D. C. (2014). A comparison study of validity indices on swarm-intelligence-based clustering. *IEEE Transactions on Systems Man & Cybernetics Part B*.

[37] Schro F., Kalenichenko D., Philbin J. A unified embedding for face recognition and clustering.

[38] Liu H., Cheng J., Wang F. (2018). Sequential subspace clustering via temporal smoothness for sequential data segmentation. *IEEE Transactions on Image Processing*.

[39] Judd K., Small M. (2000). Towards long-term prediction. *Physica D: Nonlinear Phenomena*.

